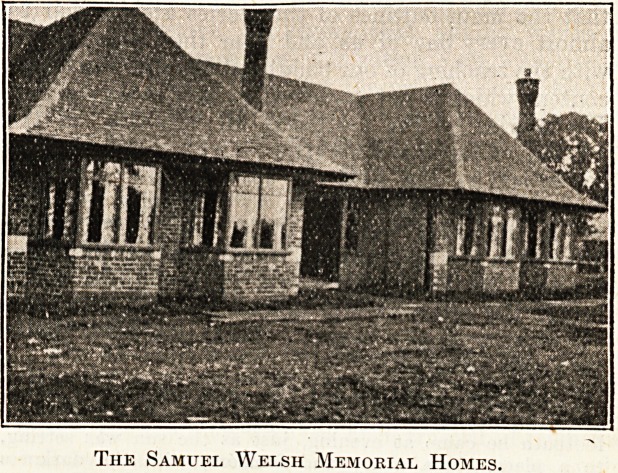# Homes for Aged Nurses

**Published:** 1923-11

**Authors:** 


### HOMES FOR AGED NURSES.

A HOME for Aged and Incapacitated Nurses, erected in memory of the late Samuel Welsh, was opened recently at Knowle, in Warwickshire. Mr. Welsh was the founder and secretary of Walsall Hospital with which Sister Dora was so long connected, and the land upon which the Home has been built was given by Mr. Frederick Slater, whose wife is a daughter of the late Mr. Welsh. The Home stands amid delightful surroundings, and the cost of erection was £1,360. Alderman J. A. Watson, in declaring the Home open, referred to the necessity for the provision of suitable places where those who had sacrificed the best years of their lives in ministering to the needs of suffering humanity could spend their old age in comfort. He hoped that these Homes would be extended, and many more built all over the country.

The Home is one large building, erected after the style of a bungalow, with loggia in the centre, and in

THE SAMUEL WELSH MEMORIAL HOMES.

each corner is a suite of rooms for each nurse, composed of living-room, bedroom, larder and scullery. There are four suites of rooms, to accommodate four nurses. From each living-room there is communication to a common corridor, from which there is access to the back entrance, tool room, and bathroom. Each Home has its own entrance, so that the nurses may have absolute privacy if they choose. The Homes are free of rates and taxes, and it is hoped in the future to grant gas and coal. Curtains have been provided, but nurses must find their own furniture and have 10s. per week income. The Homes are not yet quite finished, but the outside painting and gardens are being pushed forward at the best possible speed, and the building should be ready for occupation in a week or two. The Committee will consider applications if they are sent in at once. They should go to Mr. Victor A. Bailey, Green Bank, Knowle, Birmingham.

### The Welfare of Miners.

As the result of the Miners' Welfare Fund, which has been formed by a levy of 1d. per ton of coal mined, many useful schemes for the improvement of social conditions in mining areas are now in progress. Convalescent homes, recreation institutes, sports grounds, pit-head baths and educational facilities are among the benefits of the fund. A special section on "Miners' Welfare" is now a monthly feature of *Industrial Welfare*, the official organ of the Industrial Welfare Society.

## Figures and Tables

**Figure f1:**